# Commentary: The analyst and automation

**DOI:** 10.1155/S1463924679000687

**Published:** 1979


### Commentary

### The analyst and automation

That the implementation of automation is a systems problem and must involve a multidisciplinary approach has been stressed many times in this journal. The skills required to achieve effective automation inlude electronics, statistics, computer expertise, combined with business and organisational acumen. Indeed, there may, in the past, have been too much input from the experts in mini and micro computers and not enough consideration of business and organisational factors. This may be a daunting prospect for the traditional analytical chemist. But the analyst's role in this process is nonetheless vital. However, it would seem that analysts have been ill prepared for the task and do not fully appreciate or recognise their responsibilities. In the age before automation the analyst's responsibilities were, perhaps, more clearly defined. Boundaries were clear-cut; samples were received in the laboratory and by using a set of well established manual chemistries a set of results were delivered to the 'client'. Now, with even a small element of automation, it is far too easy for the analyst to blame a failure in his procedures on the instrumentation. If an instrument is not working, is unreliable or is unsuitable for the task no one person can take the blame. It is necessary to encourage a collective responsibility and the analyst must be an integral part of the team. He cannot sit back and wait for management, electronics experts and/or instrument manufacturers to provide him with an automated means of finding results.

### The analyst's job down-graded?

It is sometimes thought that the analyst's job has been down-graded by the introduction of automation and computerisation. It is believed that his hard earned skills and experience are being replaced by the requirement merely to operate an instrument which overrates his capability of responsible judgement. He may also feel less able to substantiate results he has obtained from an automatic instrument than he could from his manual procedures. This may be particularly so if he has not had a hand in purchasing the instrument.

No! The analyst's job has definitely not been down-graded by the introduction of automated systems. In a laboratory dedicated to automation the analyst holds a central, important position in the team, although it may well be different from the accepted role of a traditional analyst. He retains ultimate responsibility for the status and quality of the results produced by his laboratory. He must therefore cooperate fully with his in-house electronics engineer and/or the manufacturer of his instrumentation to acquire an understanding of the principles and operation of his machines. In this way he can best use their advantages and be aware of their disadvantages and short-comings. He will come to appreciate that if an instrument is working to specification in all aspects, the limiting factor in the quality of results will usually be the reliability of the chemistry of the method being used. In this situation the analyst has not conceded responsibility for the methodologies; he can use his knowledge and experience to explore the potential of an instrument by modifying the analytical method to be more compatible with the instrument design or modifying the chemistry to suit the instrumentation.

### Instrument specification

A further responsibility of the analyst is in the specification of the requirements for the performance of any automatic instruments which may be purchased or constructed for his use. This is a new and unfamiliar role for the analyst which he can only discharge effectively with the active cooperation of colleagues experienced in other disciplines. The alternative is to take manufacturers' literature at face value; a course which is fraught with pitfalls and one not to be encouraged.

In this area some interesting progress is expected from the clinical chemists. The International Federation of Clinical Chemists Expert Panel on Instrumentation are about to publish guide lines for the specification and purchasing of instruments which, of course, largely involves automatic instruments.

### Management requirements

While a specification must primarily consider the analyst's needs, it must also take into consideration the management requirements for quality control, cost effectiveness etc, and the ultimate 'customers' requirements from the analyses. In other words, the specification should include all aspects of the analytical process and not be limited to the function of measurement. It should include management requirements as well as data processing, archiving and reporting as integral aspects of automation. The instrumentation ultimately chosen to meet the specification must not restrict any of the people involved in their respective tasks. Very often the analyst may feel that he is being restricted because the computer system will not provide what he asks for. If his request can be justified and can be dealt with economically then the system should be flexible enough to meet it.

### Education

Problems in implementing the above fundamentals often arise through a lack of communications or through education difficulties. Professor Malmstadt highlighted the need for educational courses in the April issue of this Journal (page 119) and in this issue the equally important question of who to educate is discussed by Annett (page 241) and Young (page 243). The answer perhaps is that education is required at all levels. If one holds to the fundamental tenet that automation involves a multidisciplinary approach then education in automation should not be confined to the analyst. Just as the analyst must concern himself with management considerations such as economics and cost effectiveness, so managers must be acquainted with the analyst's requirements and problems. It is particularly important for all concerned to be conscious of the cost of instruments relative to staff costs. In some situations what the analyst visualises as a simple modification to software or hardware may well result in a considerable amount of work for a computer specialist. The cost of this work may well result in the modification being shelved.

An interesting educational experiment is reported in this issue (page 249). The Summer School on Automatic Analysis was attended by personnel representing the full spectrum of jobs associated with analytical chemistry. In this school a real world course problem studied in group discussion provided the desired cross-fertilisation with each person in the group gaining some appreciation of the others problems.

### Communication

With a range of disciplines being involved with automation, communication becomes a real problem; each discipline has its own jargon which seems to fight against effective communication across interdisciplinary boundaries. The analyst may present to an instrument company or a systems designer a scheme for the solution of a problem. This may reflect how the analyst sees the problem being solved and his limited experience of automation.

Now more than ever instrument companies woo potential customers with the claim that their instruments are the panacea for all analytical problems. Often this claim is associated with the introduction of microprocessor technology. The unsuspecting analyst may find that he has purchased an instrument which is more sophisticated than he needs; he cannot be expected to predict that the instrument will meet any future requirements. If he has not been fully involved in all stages of purchase he may find himself with an instrument which is incapable of the requirements to be placed upon it.

It cannot be stressed too often that an essential requirement is a full and detailed specification of the analytical needs. The specification should be assessed by the analyst, and systems designer/manufacturer in cooperation. A solution meeting this specification will then be designed which makes use of all the available resources including in-house and commercial technical and economic considerations. Only in this way can new technology be effectively and economically introduced.

The analyst has an important role in the implementation of automation. It is clearly not a simple role. It requires a commitment to the overall objectives of automation and the involvement and encouragement by managment. A willingness to transpose ideas across disciplinary boundaries is an essential requirement for all concerned. The most difficult constraint to overcome in the introduction of successful automation is a proper understanding of the chemistry involved and correct use of materials of construction.

This journal provides a medium for the discussion of automation problems and articles in it will hopefully overcome the barriers to automation. Recently the symposium 'Analysis 1979' brought together clinical and industrial chemists to discuss papers of mutual interest. It formed a valuable exchange of ideas and philosphies and it is hoped that future meetings will be organised along similar lines. The papers presented at this meeting had considerable merit and for future occasions it is hoped that a larger audience can be attracted.

*Peter B. Stockwell*

### Education for automation–reaching the right people?

In the April issue of this journal, Professor Howard V. Malmstadt presented a commentary on the problem of education in automated analysis. As an analyst who was trained in the classical methods and had to learn automation techniques by laboriously extracting material from a variety of journals and other sources, I have no quarrel with his contention that an integrated program of education is sorely needed in the training of automatic analysis as part of advanced degree programs. However, I believe that it overlooks a crucial but parallel point: the acceptance of automation for routine laboratory work will not ultimately depend on these people, but instead on others whose scientific training is considerably less than the Ph.D. In the specific case I wish to discuss, the hospital/clinical setting, the people having the most influence on automation decisions will belong to one of two groups, administrators and laboratory technicians. To my knowledge, no training programs appropriate for either group exist anywhere.

The small doctor's office or clinic is not important here because the number of blood, urine, and other samples processed is small enough to be conveniently handled by non-automated techniques. The large hospital, however, is a different story. A typical 500 bed hospital will process upwards of 20,000 blood samples each year, and the number of urine samples will be similar. Clearly, automated analysis techniques are suggested in order to handle the sheer volume of samples, yet few hospitals have anything more automated than a sample changer in their laboratory. When older equipment wears out, it is replaced by similar non-automated instruments rather than by more modern automated ones. The laboratory is thus crowded with technicians, who must work feverishly to keep up with the work load. The failure of any instrument is a disaster, as there are seldom spares, and upon repair many hours of overtime are required for the technicians to catch up. If for any reason the work load increases, only one solution is considered – hire more technicians.

This situation is perpetuated by the hospital administration, whether it be the medical personnel or the business personnel. First, the cost vs. benefit of automated analysis has never been explained to them. They see high price tags on automated instruments but do not realize what beneficial change the instruments would effect on the clinical lab. University-style classes will not help these people, as most have neither the advanced technical background nor the time required to digest such a course. Short courses, seminars, and continuing education classes are desperately needed to fill this gap. Such courses presently do not exist, nor does literature at a sufficiently non-technical level that a course could be built around it. Courses of this type would also help alleviate a second problem, namely that many administrators still consider microprocessors and automated instruments as "big boy's toys" rather than practical devices and are thus reluctant to commit money for them. This problem is aggravated by the mystic vocabulary which surrounds computer devices in general.

Occasionally a hospital will be blessed with a far-sighted or well-educated administrator who can look beyond these difficulties and raise a new point: few, if any, training programs for clinical laboratory technicians include automated analysis. Thus if the hospital buys automated instruments, the technicians will not be able to operate them, and educational opportunities for them to learn how are almost nonexistent. For this same reason, the technicians themselves seldom support a change to automation even though it would make their job easier and more efficient.

Thus although the number of samples and the number of tests per sample make the hospital laboratory a logical candidate for automation, few hospitals have accepted it because it has neither support from the administrators who must pay for it nor from the technicians who would use it. This lack of support arises from a lack of education as to what automation can do.

There is no situation in industry that corresponds to this. The myriad of government agencies and regulations which affect product quality, air and water pollution, and worker health and safety have forced industrial laboratories to undertake substantial testing programs. Automation has become both the accepted and the preferred method for conducting the requisite number and type of tests to ensure compliance. The people responsible for such testing programs are usually those to whom the usual automation class is addressed, Ph.D's or others with advanced scientific training.

*Continued on page 243*

